# Characterization of a Novel Murine Colon Carcinoma Subline with High-Metastatic Activity Established by In Vivo Selection Method

**DOI:** 10.3390/ijms21082829

**Published:** 2020-04-18

**Authors:** Liqiu Ma, Yoshimitsu Sakamoto, Akinori Kanai, Hiromi Otsuka, Akihisa Takahashi, Kazuhiro Kakimi, Takashi Imai, Takashi Shimokawa

**Affiliations:** 1 National Institute of Radiological Sciences, National Institutes for Quantum and Radiological Science and Technology, Chiba 263-8555, Japan; maliqiu@ciae.ac.cn (L.M.); c.msyu44@gmail.com (Y.S.); hiromi1029ohtsuka@gmail.com (H.O.); imai.takashi@qst.go.jp (T.I.); 2 Gunma University Heavy Ion Medical Center, Maebashi, Gunma 371-0034, Japan; a-takahashi@gunma-u.ac.jp; 3 China Institute of Atomic Energy, Beijing 102413, China; 4 Graduate School of Science, Toho University, Funabashi, Chiba 274-8510, Japan; 5 Department of Molecular Oncology and Leukemia Program Project, Research Institute for Radiation Biology and Medicine, Hiroshima University, Hiroshima 739-8527, Japan; akkanai@hiroshima-u.ac.jp; 6 Department of Immunotherapeutic, The University of Tokyo Hospital, Tokyo 113-8655, Japan; kakimi@m.u-tokyo.ac.jp

**Keywords:** metastasis, pre-metastatic niche, immune-response, immunosurveillance, in vivo selection

## Abstract

The establishment of cancer cell lines, which have different metastatic abilities compared with the parental cell, is considered as an effective approach to investigate mechanisms of metastasis. A highly metastatic potential mouse colon cancer cell subline, Colon-26MGS, was derived from the parental cell line Colon-26 by in vivo selection using continuous subcutaneous implanting to immunocompetent mice. To clarify the mechanisms involved in the enhancement of metastasis, morphological characteristics, cell proliferation, and gene expression profiles were compared between Colon-26MGS and the parental cell. Colon-26MGS showed over 10 times higher metastatic ability compared with the parental cell, but there were no differences in morphological characteristics and in vitro proliferation rates. In addition, the Colon-26MGS-bearing mice exhibited no marked change of splenocyte population and lung pre-metastatic niche with tumor-free mice, but there were significant differences compared to Colon-26-bearing mice. RNA-seq analyses indicated that immune costimulatory molecules were significantly up-regulated in Colon-26MGS. These results suggest that Colon-26MGS showed not only higher metastatic activity, but also less induction property of host immune response compared to parental Colon-26. Colon-26MGS has proven to be a novel useful tool for studying multiple mechanisms involving metastasis enhancement.

## 1. Introduction

In recent years, considerable advances have been made in the research and treatment of cancer [1]. However, cancer is still the first leading cause of death in Japan and the second leading cause of death in the world [2,3]. Early-stage cancer may be cured by surgery and radiotherapy, but once cancer develops to later stages spreading to other organs, even if the primary tumor receives effective treatment, distant metastasis becomes the crucial issue for prolonging patient survival. In fact, distant metastasis is responsible for approximately 90% of cancer-related deaths [4]. Therefore, understanding of cancer metastatic mechanisms is vital for clinical diagnosis and targeted therapy, leading to improved survival of patients.

Comparing the characteristics of tumors with metastatic potential from different patients is known to be an efficient way to understand the mechanisms underlying metastasis. By this approach, several important genes, which showed different expression levels between tumors with various metastatic potentials were identified [5,6,7]. However, there are difficulties comparing cancer cell lines established from different patients, because of large individual differences. On the other hand, cancer cell lines with a highly metastatic potential can be easily compared with the parental cell line, and metastasis-related genes can be identified because these are sub-clones from the same origin [8,9]. In vivo selection, which is repetitive isolation of metastatic cells and re-implantation using a tumor-bearing mouse model, is an efficient method used to establish cancer cell lines with highly metastatic potential. Characterization of these in vivo selected cells reveal not only highly metastatic ability but also altered expression of well-known metastasis-related genes. A large number of metastasis researches have been carried out using various established cancer cells and have identified numerous novel metastasis-related genes, including *AGGRUS*/*PDPN*, *CDC42*, and *VEGF* [10,11,12].

“Avoiding immune destruction” has recently become acknowledged as a novel hallmark of cancer, and is now known to be related to cancer progression [13,14]. The abilities of cancer cells to edit/modify tumor immunity or escape from tumor immunity are essential requirements for aggressive cancer. Higher immunogenic cancer cells enhance the population of immunosuppressive inflammatory cells such as MDSCs (myeloid-derived suppressor cells) and Tregs (regulatory T cells), then they inactivate CTL (cytotoxic T lymphocyte), and finally escape immunosurveillance [15,16]. This novel hallmark is a result of the interaction between the systemic immune environment and tumor cells. However, it is still not clearly understood how it contributes to metastasis enhancement.

In the current study, we established a colon cancer cell line with a high metastatic potential, named Colon-26MGS, (Metastatic Gao State, “Gao” means “high” in Chinese), by in vivo selection and investigated the mechanism of tumor metastasis.

## 2. Results

### 2.1. Characterization of a Novel Highly Metastatic Cancer Cell Line Colon-26MGS

**Figure 1:**
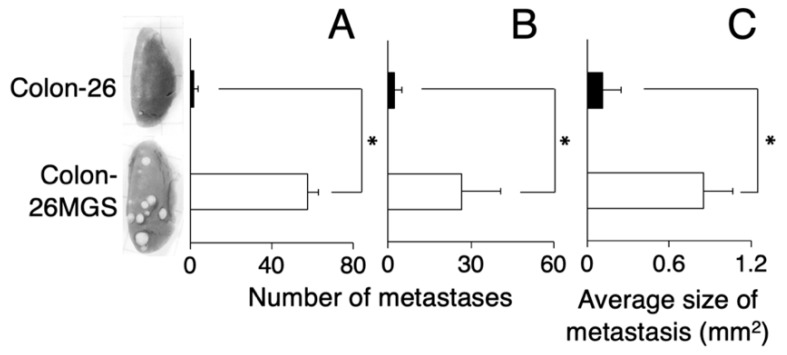
Comparison of metastatic ability between Colon-26MGS and Colon-26. Number of lung metastasis by subcutaneous injection (**A**) and by intravenous injection (**B**) of cancer cells, and size of lung metastasis nodules (**C**) of cancer-bearing mice. Five mice were analyzed per group. * *p* < 0.05 by Student’s *t*-test. Bars indicate standard error calculated using data from three independent trials.

To verify the established cell line as a highly metastatic subline, we counted the number of lung metastatic nodules of subcutaneously transplanted mice. At day 21 after transplantation, there were over 10 times more pulmonary metastasis nodules for Colon-26MGS cells compared with the parental cell Colon-26 (Figure 1A). In addition, the metastatic potential of the Colon-26MGS cells were evaluated by an intravenous implant of this cell. A 12.2-times higher number of pulmonary metastasis nodules was observed for Colon-26MGS compared to Colon-26 cells (Figure 1B). Furthermore, after subcutaneous transplantation, the sizes of lung metastasis nodules of Colon-26MGS-bearing mice were 7.7 folds larger than Colon-26 (Figure 1C).

**Figure 2:**
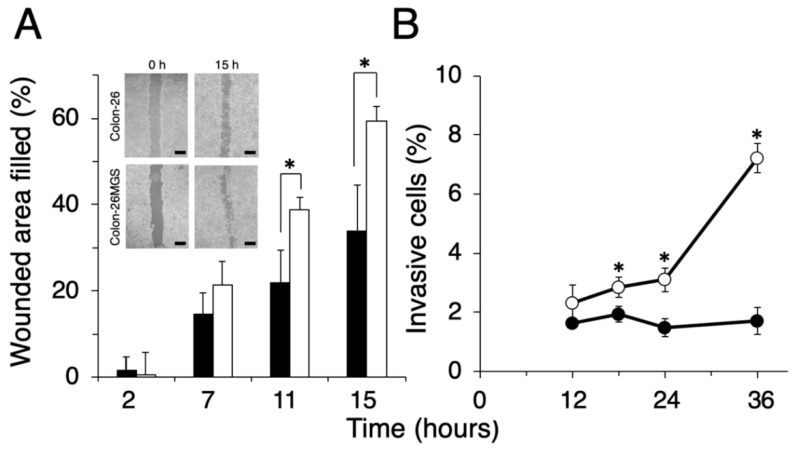
Comparison of migration and invasion ability between Colon-26MGS and Colon-26. (**A**) Filled wounded area of cancer cells (scale bar: 1 mm); (**B**) Invasion ability. Colon-26 (closed symbols), Colon-26MGS (open symbols) * *p* < 0.05 by Student’s *t*-test. Bars indicate standard error calculated using data from three independent trials.

**Figure A1:**
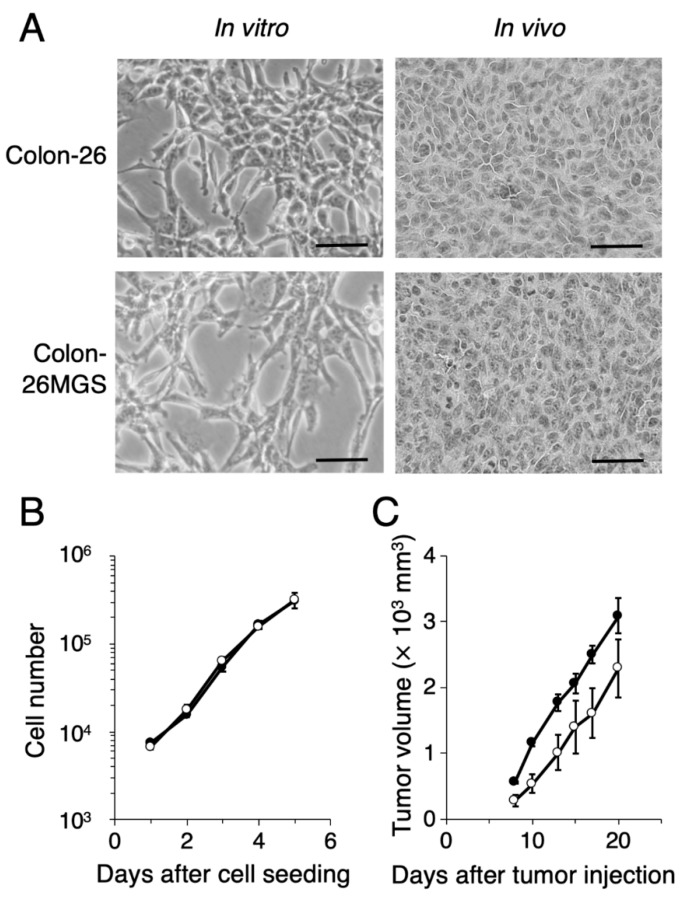
Phenotypic characterization of a novel highly metastatic cancer cell line Colon-26MGS. (**A**) In vitro and in vivo tumor cell morphology (scale bar: 50 μm). (**B**) In vitro cancer cell and (**C**) in vivo primary tumor cell proliferation of Colon-26 (closed circles) and Colon-26MGS (open circles). Bars indicate standard error calculated using data from three independent trials.

To evaluate other general metastasis-related abilities, migration and invasion assays were performed. Wound healing assay demonstrated that filled wounded area of Colon-26MGS was 1.8 or 1.7 times larger compared to that of parental Colon-26 cells observed at 11 h or 15 h after wounding, respectively (Figure 2A). In addition, higher invasion ability of Colon-26MGS cells were observed from 18 h to 36 h after cell seeding (Figure 2B). On the other hand, although no significant differences in morphological features (Figure A1A) and in vitro proliferation rates (Figure A1B) between Colon- 26 and Colon-26MGS cells, there were observed differences between the primary tumor volumes after injection of Colon-26 and Colon-26MGS (Figure A1C). These results showed that Colon-26MGS exhibited metastatic potential in mice, although it is similar in morphological and in vitro proliferation characteristics with the parental cell line.

### 2.2. Evaluation of Cancer-Related Features in Colon-26MGS-Bearing Mice

**Figure 3:**
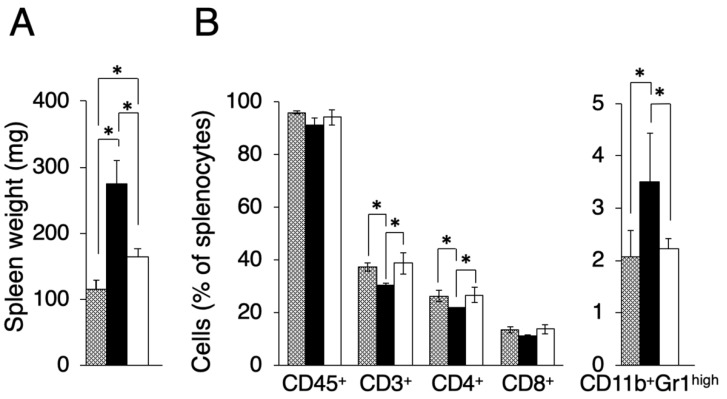
Evaluation of splenocytes in Colon-26MGS and Colon-26 bearing mice. Splenocyte populations were evaluated in tumor-free (gray column), Colon-26 (close column) and Colon-26MGS (open column) bearing mice. (**A**) Spleen weight; (**B**) Leukocytes, Total T cell, Helper T cell, and Killer T cell, MDSCs were evaluated as CD45^+^, CD3^+^, CD4^+^, CD8^+^, CD11b^+^Gr1^high^ cells. * *p* < 0.05 by Student’s *t*-test. Bars indicate standard error calculated using data from three independent trials.

**Figure A2:**
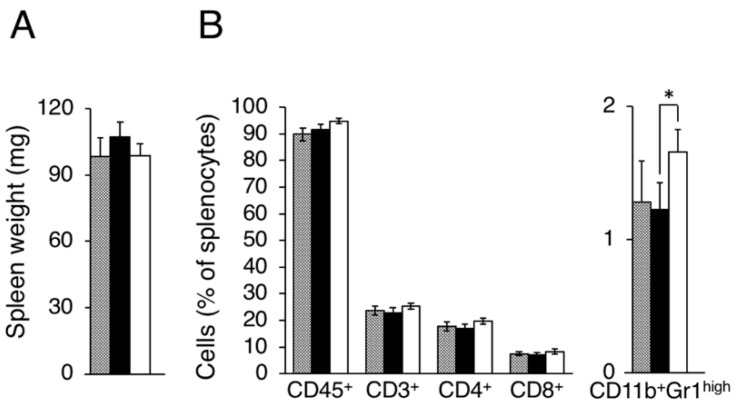
Evaluation of splenocytes in LM8 and Dunn bearing mice. Splenocyte populations were evaluated in tumor-free (gray column), Dunn (closed column) and LM8 (open column) bearing mice. (**A**) Spleen weight; (**B**) Leukocytes, Total T cell, Helper T cell, and Killer T cell, MDSCs were evaluated as CD45^+^, CD3^+^, CD4^+^, CD8^+^, CD11b^+^Gr1^high^ cells. * *p* < 0.05 by Student’s *t*-test. Bars indicate standard error calculated using data from three independent trials.

In the course of evaluating other effects by transplanted tumors, we found that the spleen size differed between Colon-26MGS and Colon-26 bearing mice. It is known that there is a tendency for spleen size to increase in tumor-bearing mice compared to näive mice [17]. As shown in Figure 3A, the spleen weight of Colon-26 and Colon-26MGS bearing mice were significantly increased than näive mice, although the increase observed in Colon-26MGS was not as great as in Colon-26-bearing mice. To investigate in more detail, we measured the population of splenocytes. In line with the results of spleen weight change, splenocyte population, especially total T cells (CD3^+^ cells), and helper T cells (CD4^+^ cells) changed significantly by transplantation of Colon-26 cells, but not by transplantation of Colon-26MGS cells (Figure 3B). To verify whether the distribution pattern of spleen immune cells in Colon26MGS-bearing mice is universal, we analyzed the splenocytes in Dunn-bearing mice and its highly metastatic subline LM8-bearing mice. In line with the results for Colon-26MGS, there were no marked changes in spleen weight and population of CD3^+^, CD4^+^, CD8^+^ T cells by transplantation of high metastatic potential LM8 cells, which was sorted by the in vivo selection method (Figure A2). In addition, the population of CD11b^+^Gr-1^high^ myeloid cells, which is known as myeloid-derived suppressor cells (MDSCs) [18], were increased in Colon-26-bearing mice, but not in Colon-26MGS-bearing mice (Figure 3B). Although LM8-bearing mice showed higher MDSCs population than Dunn-bearing mice, there are no marked changes of MDSCs by transplantation of either Colon-26MGS or LM8-bearing (Figure A2B). These results indicated that the Colon-26MGS cells have a weaker impact on the host immune responses compared to parental Colon-26 cells.

### 2.3. Up-Regulated Expression of Immune-Related Genes in Colon-26MGS

To further understand the differences between Colon-26MGS and Colon-26 cells especially immune-related differences, gene expression profiles of the cells were measured by RNA-seq analysis and GO enrichment analysis was performed. To screen causative gene expression changes for higher metastatic ability, we additionally included the gene expression profile of Colon-26HM4 cells, established at the four-times repetition of metastases implanting which did not show enhanced metastatic ability. 752 genes and 253 genes were selected as Colon-26MGS specifically up- or down-regulated genes, respectively. Table A1 and Table A2 show the results of the GO enrichment analysis for the up- or down-regulated genes, respectively. Contrary to our expectation, metastasis-related GO categories were not listed in the up-regulated GO categories. Interestingly, immune-related GO categories, specifically CD40 receptor complex (GO:0035631), were selected by GO analysis of the up-regulated gene.

To confirm the gene expression profile, we analyzed the expression of selected genes by the quantitative reverse transcription-polymerase chain reaction (qRT-PCR). In both next-generation sequencing (NGS) and qRT-PCR analysis, immune-related genes *CD40*, *CD11c*, *CD80*, and angiogenesis-related gene *VEGFA* were up-regulated in Colon-26MGS cells (Table 1 and Table 2). On the other hand, invasion related genes *MMP2*, *MMP9*, and angiogenesis-related placental growth factor (*PGF*) gene expressions were down-regulated or remained the same in the Colon-26MGS cells. On the other hand, in the highly metastatic LM8 subline, which was established by the in vivo selection method in the previous study, the expression of metastasis-related genes but not immune-related genes, were mainly changed compared to the parental Dunn cell line (Table A3). These results suggested that not only general metastatic abilities but also immune-related features could be altered in Colon-26MGS cells.

### 2.4. Comparison of Pre-Metastatic Niche Preparation between Colon-26MGS and Colon-26

**Figure 4:**
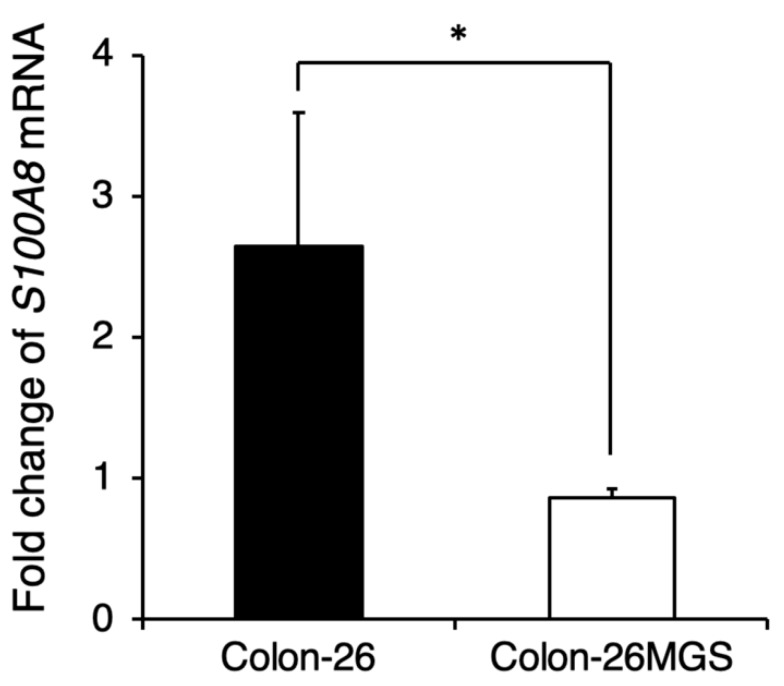
Comparison of pre-metastatic niche preparation between Colon-26MGS and Colon-26. Colon-26 or Colon-26MGS cells were transplanted into the right hind leg, and 17 days later, the lung tissue sample was collected for qRT-PCR. *S100A8* mRNA expression in the lung tissue of Colon-26MGS and Colon-26 was evaluated by qRT-PCR. * *p* < 0.05 by Student’s *t*-test. Bars indicate standard error calculated using data from three independent trials.

**Figure A3:**
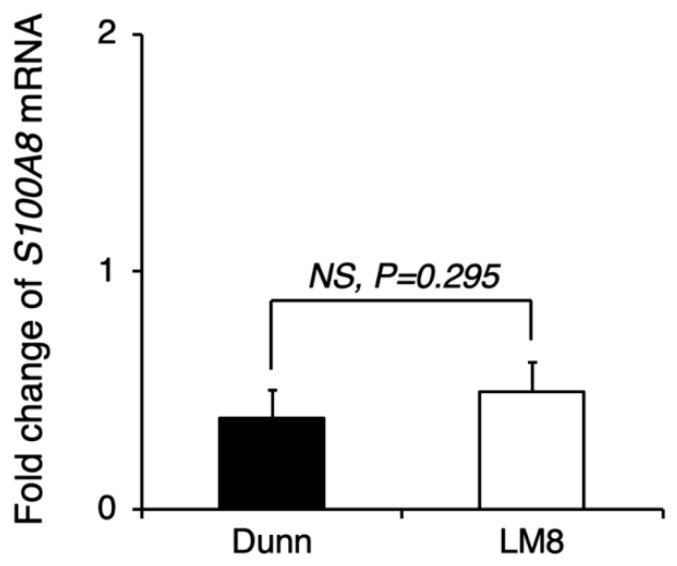
Comparison of pre-metastatic niche preparation between LM8 and Dunn. LM8 or Dunn cells were transplanted into the right hind leg, and 17 days later, the lung tissue sample was collected for qRT-PCR. *S100A8* mRNA expression in the lung tissue of LM8 and Dunn was evaluated by qRT-PCR. NS, no significant differences, by Student’s *t*-test. Bars indicate standard error calculated using data from three independent trials.

Although gene expression analyses indicated changes in immune-related gene expressions, the effects of cell implantation on the host immune response are still unclear. To evaluate the involvement of immune reactions in the metastatic process we investigated whether the pre-metastatic niche preparation was changed in Colon-26MGS by qRT-PCR. In Colon-26-bearing mice, S100A8 expression in the lung, which is known as the marker of the pre-metastatic niche [19], was drastically increased. Interestingly, even though Colon-26MGS had higher metastatic abilities, there was no change observed for *S100A8* expression in the lungs by transplantation compared to tumor-free mice (Figure 4). In LM8-bearing mice, *S100A8* expression in the lungs also showed no marked change compared to parental Dunn-bearing mice and tumor-free mice (Figure A3). These results indicated that unlike the parental Colon-26, Colon-26MGS does not induce the formation of a pre-metastatic niche, which is cancer cell-induced inflammation on the target organ.

## 3. Discussion

In the present study, a novel high metastatic potential cancer cell line Colon-26MGS was successfully established by repeated subcutaneous injection into the immunocompetent mouse. Like its parental cell line Colon-26, Colon-26MGS may be a heterogeneous cell line of different metastatic potential clones [20]. Our results demonstrated that the Colon-26MGS cell line exhibited not only enhanced metastatic activity but also less stimulation of host immune responses. To our knowledge, this is the first report using a newly established cell line to show the enhancement of metastasis with lower immune response activation capacity. This new highly metastatic Colon-26MGS cell line has proven to be a useful tool for studying multiple mechanisms involving metastasis enhancement.

Metastasis is a complex, multistep process involving migration from the original local tumor, invasion through the extracellular matrix barrier, passing through the circulation system, and finally arriving at the distant target tissue. Analysis of high metastatic potential cancer cells is an effective approach for exploring the unknown mechanisms of metastasis. Generally, the metastatic potential is considered to be correlated with enhanced cancer cell migration and invasion abilities [21,22]. Epithelial-mesenchymal transition (EMT) is also a critical step of metastasis approach. We analyzed the NGS data of EMT-related genes and found that although *CDH1* showed low gene expression in both Colon-26MGS and Colon-26, *DDR2*, *SNAI1* and *TWIST2* showed a higher expression in Colon-26MGS (Table 1). These results suggest that EMT may be involved in the Colon-26MGS metastasis enhancement mechanism. Other unique gene expression changes associated with metastatic phenotypes, such as angiogenesis inducer vascular endothelial growth factor A (*VEGFA*) [12], platelet-aggregating factor podoplanin (*PDPN*) [10], the direction of migration-related *CDC42* [23] and invasion associated matrix metalloproteinase-2 (*MMP-2*) [24] were also identified. In this research, we established the Colon-26MGS subline with enhanced metastatic abilities, such as migration and invasion (Figure 2), which also exhibited higher gene expression of previously reported metastasis-related genes, such as angiogenesis-related *VEGFA* and hematogenous metastasis-related *PDPN* (Table 1 and Table 2). On the other hand, increased expression was not observed for migration-related *CDC42* and invasion-related Matrix metalloproteinase-2 (*MMP2*) [8,11]. These results indicated that some elements of the enhanced metastatic ability of Colon-26MGS might be contributed by angiogenesis and platelet aggregating mechanisms, which were reported previously.

We chose to transplant cells ectopically due to technical limitations. However, this method may not be the best model for relevant microenvironmental-cell interactions, as gene expressions in cells, including cancer cells, are influenced by the surrounding microenvironment. When Colon-26MGS cells were being established, it is undeniable that the impact of the tumor microenvironment on the metastatic process will not be the same for ectopic transplantation and orthotopic transplantation. Due to the different types of intra-tumoral local tumor cells and infiltrated immune cells in the microenvironment, different cell–cell interactions result. In addition, liver metastasis is extremely common in human colorectal cancer patients, with liver metastasis occurring in 20% to 25% of patients at the time of initial diagnosis, but no liver metastasis has been observed in neither Colon-26MGS nor parental Colon-26-bearing mice. Therefore, the changes observed in the Colon-26MGS may be because these cells have been interacting with the subcutaneous microenvironment, and not their usual colon microenvironment, and thus these changes may not accurately model/represent that found in colon cancer cells in human patients that have metastasized to the lung. No matter how, our result is considered to be a contribution at least to understanding the universal metastatic steps that are outside of the microenvironmental-cell interactions.

It became clear that the immune system is an important factor for metastasis formation [25]. Along with cancer progression, specific antigens, including neoantigens are secreted from the cancer cells due to spontaneous mutation [26]. In the immune system, dendritic cells can be used to trigger a response against cancer cells. Specific tumor antigens are introduced directly into the dendritic cells, then tumor antigens are presented to T cells, activating them and directing killer T cells to find and destroy cancer cells [27]. Concomitant immunity suppresses the adhesion ability to destination organs of circulating tumor cells (CTCs), which get separated from the primary tumor, thus inhibiting metastasis formation [28]. However, a small number of CTCs can escape from immunological surveillance and become widespread to form metastatic cancers [13,29]. Therefore, it is clear that the host immune response has a significant impact on metastasis formation, but the molecular mechanisms are still not well understood. The in vivo selection method using the immunocompetent mouse model is useful not only for the screening of cancer cells with higher migration and invasion potentials but also has the potential to filtrate the survivors from immune system attack. In general, changes in splenocyte population and lung pre-metastatic niche formation are observed in a tumor-bearing immunocompetent mouse model, including the Colon-26-bearing mice. In contrast, the Colon-26MGS-bearing mice model were similar to tumor-free mice. These results indicated that there are significant differences in the host immune response after implantation between the Colon-26MGS cells and the parental Colon-26.

It is well known that the costimulatory protein CD40 and CD80 molecules, which bind to helper T cell ligands, are generally expressed on B cells, macrophages, and dendritic cells, but only rarely expressed on cancer cells. Batrla et al. demonstrated that T cell functions are down-regulated by CD40-expressing carcinoma cells [30]. It also has been reported that the interaction between CD80 and CTLA4 generates T cell functional inactivation [31]. In addition to the anti-phagocytic effect of cancer cells by macrophages, “don’t eat me” signaling CD47 [32,33] gene expression also significantly increased in Colon-26MGS. In contrast, the expression of “eat me” signaling calreticulin (CALR) [34] was decreased in the high metastatic potential cell (Table 1). Therefore, Colon-26MGS might escape immunosurveillance by using multiple mechanisms, which act as a crosstalk between cancer cells and immune cells. These characteristic genes may be involved in the metastasis enhancement of Colon-26MGS. To our knowledge, among the high-metastatic cell lines established by in vivo selection method, Colon-26MGS is the first cell line showing features of higher immune costimulatory molecules involving metastasis enhancement and may provide new insight into the mechanisms underlying metastasis. Other reports revealed that the CD40/CD154 signaling pathway was a key factor for metastasis-related transforming growth factor-beta (*TGF-beta*) production in cancer cells [35,36] and also for matrix metalloproteinases secretion in aorta and podocytes [37,38]. In our results, *CD40* expression was significantly higher in Colon-26MGS than parental Colon-26 (Table 1 and Table 2). Moreover, TGF-beta receptor II (*TGFBR2*) was also increased in Colon-26MGS (Table 1) but no increase in *MMPs* expression was observed (Table 1 and Table 2). This could be the cause of CD154 absence in the in vitro condition. However, the high expression of CD40 might be able to promote metastasis by locally secreting MMPs and TGF-beta in the in vivo condition if CD154 is abundantly expressed in the microenvironment.

Furthermore, we have examined the gene expression in tumors by qRT-PCR. Compared to Colon-26, not only *MMPs* but also T cell surface marker expression, such as *CD4* and *CD8a*, had increased on Colon-26MGS tumor (Table A4). Therefore, the high metastasis ability of Colon-26MGS is likely related to the increased *MMPs* in the tumor, which may be produced by intratumoral infiltrated immune cells.

Interestingly, Colon-26MGS-bearing mice show larger lung metastasis nodules compared to parental cells (Figure 1C). As there was no marked change in morphology and proliferation ratio between Colon-26MGS and parental Colon-26, this phenomenon did not depend on morphology and proliferation at the target organ (Figure A1). For another, the higher migration and invasion ability of Colon-26MGS indicated the possibility of Colon-26MGS entering the bloodstream earlier than Colon-26, thus, CTCs can reach the metastatic target organ earlier. In addition, metastatic latency, which is regulated by the immune system, is considered to be related to the metastasis size [39]. It has been reported that high metastatic potential osteosarcoma LM8 shows 1 to 2 weeks of reduced latency in the metastatic niche [8,40]. Therefore, the larger lung metastasis nodules observed in Colon-26MGS-bearing mice also could be the result of faster access to the metastatic target organ, and/or reduced metastatic latency in the target organ.

The metastatic enhancement of Colon-26MGS was acquired by various changes involving complex metastasis steps. Our results showed it is possible to establish higher metastatic potential cell lines with not only enhanced general metastatic features but also with the ability to evade the host immune system by the in vivo selection method using immunocompetent mice. The novel highly metastatic Colon-26MGS cell line has proven to be an efficient tool for investigating the multistep mechanism of metastasis.

## 4. Materials and Methods

### 4.1. Mice and Cancer Cell Lines

Six-week-old female BALB/c and C3H/He mice were obtained from Japan SLC Co., Ltd. (Shizuoka, Japan). All protocols for animal experiments were reviewed and approved by the National Institute of Radiological Sciences Institutional Animal Care and Use Committee. Colon-26 murine colon carcinoma cell lines and LM8 murine osteosarcoma cell lines were purchased from Riken BioResource Center (Tsukuba, Japan). Dunn murine osteosarcoma cells were a kind gift from Dr. Kazuyuki Itoh (Osaka Medical Center for Cancer and Cardiovascular Disease, Osaka, Japan). Colon-26, Colon-26MGS and Colon-26HM4 cells were cultured in RPMI1640. Dunn and LM8 cells were grown in DMEM with high glucose. All media contained 10% fetal bovine serum, penicillin (50 units/mL), and streptomycin (50 μg/mL). All cells were maintained at 37 °C in an incubator containing 5% CO_2_.

### 4.2. Establishment of Colon-26MGS

**Figure 5:**
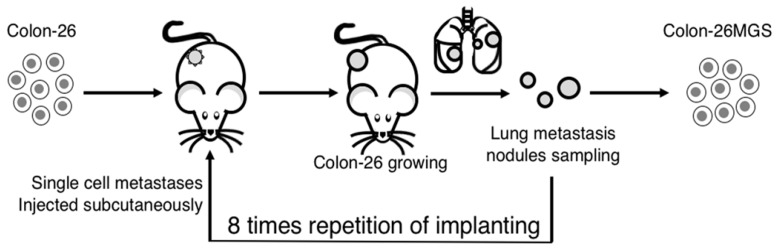
Illustration of the continuous implanting method. Colon-26 cells were grown subcutaneously in right hind paws of BALB/c mice. One month later, the mouse was sacrificed and lung metastases nodules were collected. The metastatic nodules were digested into single-cells and injected again into naïve BALB/c mice in the same manner. After eight repetitions of this process, a highly metastatic Colon-26MGS subline was established.

To establish highly metastatic cell lines, we used the continuous implanting method. Colon-26 cells were implanted subcutaneously into the right hind leg of a BALB/c mouse. One month later, lung metastases nodules were collected and digested into single cells, as described previously [8]. The cells were implanted again into naïve BALB/c mice in the same manner. After the process was repeated four or eight times, the metastatic cancer cell, termed Colon-26HM4 or Colon-26MGS respectively, were separated and then cultured in vitro (Figure 5).

### 4.3. Tumor Growth

After subcutaneous injection of 5 × 10^5^ tumor cells into the right hind leg of the mice, tumor size was measured every two days for 2–3 weeks with calipers (*n* = 7 per group). Tumor volume was calculated according to the following formula: (a × b × c × π)/ 6, where a, b, and c represent the three orthogonal diameters of the tumor.

### 4.4. Pulmonary Metastasis Assay

Three (for subcutaneous, 5 × 10^5^ cells/mouse) or two weeks (for intravenous, 5 × 10^4^ cells/mouse) after tumor cell implantation, bilateral lungs of the mice were initially fixed in Bouin’s solution overnight. The pulmonary metastatic nodules on the surfaces of all the pulmonary lobes were macroscopically counted. The sizes of metastases were measured using Image J software (National Institutes of Health, ver.1.46r, Bethesda, MD, USA). (*n* = 5 per group).

### 4.5. Growth Properties of Tumor Cells In Vitro

Colon-26 or Colon-26MGS (3 × 10^4^ cells/dish) were plated on 35-mm plastic dishes on day 0. Cells were counted every day for five days. Each experiment was performed in triplicate (*n* = 3).

### 4.6. Scratch Assay

Colon-26MGS and Colon-26 cells (1 × 10^6^ cells/dish) were plated on 35-mm plastic dishes. Cells were grown to confluence before the cell monolayer was scratched using a 200-μL sterile micropipette tip. Images of the wounded area were captured immediately after the scratch and at different time points using inverse microscopy (IX70, Olympus, Tokyo, Japan). The wounded area was measured using ImageJ software (National Institutes of Health). Each experiment was performed in triplicate (*n* = 3).

### 4.7. Invasion Assay

The invasion ability of cells was examined by using HTS FluroBlok^TM^ Insert containing a 6.5-mm filter with a pore size of 8 µm (BD Falcon, San Jose, CA, USA). For Matrigel coating, 50 µl of 100 µg/mL Matrigel (BD Biosciences, Bedford, MA, USA) was applied to the membrane filters of the insert. Filters were dried overnight in a laminar flow hood. Colon-26MGS cells and Colon-26 cells were pre-labeled with DiIC12(3) (BD Biosciences) at a concentration of 1.25 µg/mL for 1 h at 37 °C, 5% CO_2_. Pre-labeled cells were suspended into the serum-free RPMI1640 medium and seeded into each of the upper inserts at 2 × 10^4^ cells/well, while RPMI-1640 supplemented with 10% fetal bovine serum was added in the lower chamber, and the real-time kinetic invasion assays were carried out for 36 h. The images of total cells and invaded cells were captured at different time points using the In Cell Analyzer 2000 system (GE Healthcare, Buckinghamshire, UK). The number of invaded cells in three random fields was counted using ImageJ software. Each experiment was performed in triplicate (*n* = 3).

### 4.8. Immunohistochemical Analysis

Primary tumors were fixed in 10% neutralized formalin, embedded in paraffin, sectioned at a mean thickness of 3 mm, and stained with hematoxylin–eosin. Immunohistochemical studies were performed using a streptavidin-biotin immunoperoxidase technique as described elsewhere [41]. Sections were photographed using inverse microscopy (IX70, Olympus, Tokyo, Japan).

### 4.9. Next-Generation Sequencing

Total RNA was isolated from in vitro cultured cells, using the ReliaPrep™ RNA Miniprep Systems (Promega, Fitchburg, WI, USA) following the manufacturer’s protocol. Total RNAs were converted to sequencing libraries using the SureSelect Strand Specific RNA-Seq Library Preparation kit (Agilent Technology, Santa Clara, CA, USA), according to the manufacturer’s instructions. The libraries were sequenced using Hiseq2500 sequencer (Illumina, San Diego, CA, USA) with a single 50-bp read option. The generated sequence reads were mapped onto the mouse genomic sequence (mm10; UCSC Genome Browser) using the sequence alignment Program Eland (Illumina). Only the sequence reads passing quality filtering were used for further analysis. For each RefSeq gene, the number of mapped reads per kilobase of exon per million mapped reads (RPKM) was calculated.

The deep sequencing data from the RNA sequencing analyses have been deposited under accession numbers DRA007510, in the DDBJ (DNA Data Bank of Japan) Sequence Read Archive.

### 4.10. Gene Function Annotation

The up- and down-regulated differentially expressed genes were put into the FuncAssociate to identify overrepresented Gene Ontology (GO) categories [42]. *p* < 0.05 was set as the threshold for the analysis using the hypergeometric distribution. The logarithm of the odds ratio (LOD) larger than 1 were set as the cut-off criterion.

### 4.11. Quantitative Reverse Transcription Polymerase Chain Reaction

Total RNA was isolated from cancer cells, lung, or tumor tissues by using the Reliaprep^TM^ RNA Miniprep System (Promega, WI, USA). Isolated total RNA was reverse-transcribed using the PrimeScript RT-PCR Kit with random primers N6 (Takara Bio, Shiga, Japan). qRT-PCR was performed using the Applied Biosystems 7300 Real-Time PCR system (Applied Biosystems, Foster City, CA, USA). Three housekeeping genes, namely *ARP*, *GAPDH*, and *HPRT*, were selected as internal standards. The comparative 2^-ΔΔC^_T_ method was used to analyze the relative gene expression levels. Gene-specific primers used for qRT-PCR are listed in Table A5. Each reaction was performed in triplicate.

### 4.12. Flow Cytometry Analysis

Mouse splenocytes were collected aseptically from spleens of mice by mincing the spleen tissues in a sterile Petri plate, and the erythrocytes were lysed in lysis buffer (10 mmol/L KHCO_3_, 150 mmol/L NH_4_Cl, 10 mmol/L ethylenediaminetetraacetic acid, pH 7.4). Splenocytes were prepared from tumor-grafted mice or naïve mice. ACK (Ammonium–Chloride–Potassium) Lysing buffer was used to lyse erythrocytes. Splenocytes were stained using APC-conjugated anti-mouse CD3e antibody, APC-conjugated anti-mouse CD4, FITC-conjugated anti-mouse CD8a, APC/Cy7-conjugated anti-mouse CD11b, APC-conjugated anti-mouse CD45, and PE-conjugated anti-mouse Ly6G/Ly6c (Gr-1) (1:200, BioLegend, San Diego, CA, USA) following the manufacturer’s instructions. Living cells were assessed by using 1 mg/mL Propidium iodide (PI). Analyses of fluorescence staining were performed with a Gallios flow cytometer (Beckman Coulter, Brea, CA, USA). Data were collected and analyzed using Kaluza^®^ for Gallios-Acquisition and Flow Analysis Software (Beckman Coulter). We collected at least 10,000 viable cell events per sample in each experiment.

### 4.13. Statistical Analysis

The statistical significance of differences was tested by the use of the Student’s *t*-test. The differences between the means were considered to be statistically significant if *p* < 0.05.

## 5. Conclusions

We established a novel high-metastatic potential mouse colon carcinoma subline by in vivo selection using continuous subcutaneous implanting to the immunocompetent mouse. Unlike the parental line, this metastasis-proficient subline cells, which are increased well-known metastasis-related abilities, were demonstrating enhancement of metastasis without strongly activating systemic immune response such as changing pre-metastasis. We believe that our findings have provided a novel efficient tool for investigating the multistep mechanisms involved in metastasis enhancement.

## Figures and Tables

**Table 1:** Gene expression by next-generation sequencing (NGS).

Gene Name	RPKM of Colon-26	RPKM of Colon-26HM4	RPKM of Colon-26MGS
**Metastasis-related genes**			
*MMP2*	0.24	0.00	0.01
*MMP9*	0.01	0.04	0.01
*VEGFA*	59.34	96.08	61.03
*TGFBR2*	34.91	49.9	48.12
*CDH1*	0.00	0.00	0.02
*DDR2*	22.03	25.50	33.81
*SNAI1*	14.06	19.06	21.01
*TWIST2*	66.68	102.78	101.54
*PGF*	0.07	0.04	0.02
*FN1*	319.62	379.36	396.21
*PDPN*	13.62	53.28	62.40
*CDC42*	162.03	166.66	175.74
**Immune-related genes**			
*CD40*	0.00	0.03	1.63
*CD11c*	0.19	1.87	0.28
*CD80*	2.94	4.53	2.72
*PDL1*	0.32	0.40	0.17
*CD47*	16.88	34.43	33.91
*CALR*	856.67	718.59	741.27

RPKM: Reads per kilobase of exon per million mapped sequence reads.

**Table 2:** Gene expression by qRT-PCR.

Gene Name	ΔC_T_ of Colon-26 (Normalized by *ARP*)	ΔC_T_ of Colon-26MGS (Normalized by *ARP*)	Fold Change (Colon-26MGS)	Significance
**Metastasis-related genes**				
*MMP2*	13.31 ± 0.25	17.29 ± 1.07	0.06	*
*MMP9*	14.92 ± 2.68	14.21 ± 1.80	1.63	NS
*VEGFA*	5.39 ± 0.38	4.65 ± 0.14	1.67	*
*PGF*	15.48 ± 0.48	16.11 ± 0.94	0.65	NS
*FN1*	4.04 ± 0.17	2.58 ± 0.08	2.75	*
**Immune-related genes**				
*CD40*	ND	11.24 ± 0.09	ND	*
*CD11c*	14.87 ± 0.14	12.45 ± 0.06	5.38	*
*CD80*	10.54 ± 0.24	10.09 ± 0.09	1.37	*
*PDL1*	11.29 ± 0.42	13.17 ± 0.85	0.27	*

NS: no significant; *: *p* < 0.05; ND: not detected.

## Abbreviations

DDBJ	DNA Data Bank of Japan
MDSCs	Myeloid-derived suppressor cells
Tregs	Regulatory T cells
CTL	Cytotoxic T lymphocyte
MGS	Metastatic Gao State, “Gao” means “high” in Chinese
*PGF*	Placental growth factor
EMT	Epithelial-mesenchymal transition
*VEGFA*	Vascular endothelial growth factor-A
*PDPN*	Podoplanin
*MMP2*	Matrix metalloproteinase-2
*MMP9*	Matrix metalloproteinase-9
CTCs	Circulating tumor cells
*TGF-beta*	Transforming growth factor-beta
*TGFBR2*	Transforming growth factor-beta receptor II
*CALR*	Calreticulin
NIRS	National Institute of Radiological Sciences
HIMAC	Heavy Ion Medical Accelerator in Chiba
RPKM	Reads per kilobase of exon per million mapped reads
GO	Gene Ontology
LOD	Logarithm of the odds ratio
qRT-PCR	Quantitative reverse transcription-polymerase chain reaction
NGS	Next-generation sequencing

## Appendix B

**Table A1:** Gene function annotation list of up-regulated gene sets in Colon-26MGS, but not in Colon-26HM4.

LOD	*p*-Value	GO ID	GO Name
2.99	3.60 × 10^−7^	GO:0008424	Glycoprotein 6-alpha-l-fucosyltransferase activity
2.99	3.60 × 10^−7^	GO:0036071	N-glycan fucosylation
2.52	1.43 × 10^−6^	GO:0033578	Protein glycosylation in Golgi
2.52	1.43 × 10^−6^	GO:0046368	GDP-l-fucose metabolic process
2.29	3.56 × 10^−6^	GO:0010853	Cyclase activator activity
2.29	3.56 × 10^−6^	GO:0030250	Guanylate cyclase activator activity
2.23	5.59 × 10^−11^	GO:0060718	Chorionic trophoblast cell differentiation
2.15	7.08 × 10^−6^	GO:0030249	Guanylate cyclase regulator activity
2.04	1.23 × 10^−5^	GO:0006360	Transcription from RNA polymerase I promoter
2.04	1.23 × 10^−5^	GO:0010851	Cyclase regulator activity
2.04	1.23 × 10^−5^	GO:0031284	Positive regulation of guanylate cyclase activity
1.87	1.20 × 10^−6^	GO:0048304	Positive regulation of isotype switching to IgG isotypes
1.87	1.20 × 10^−6^	GO:0060541	Respiratory system development
1.74	3.26 × 10^−6^	GO:0010459	Negative regulation of heart rate
1.74	3.26 × 10^−6^	GO:0035631	CD40 receptor complex
1.68	2.65 × 10^−13^	GO:0021952	Central nervous system projection neuron axonogenesis
1.67	5.61 × 10^−6^	GO:0048302	Regulation of isotype switching to IgG isotypes
1.64	7.18 × 10^−6^	GO:0042511	Positive regulation of tyrosine phosphorylation of Stat1 protein
1.64	7.18 × 10^−6^	GO:0090037	Positive regulation of protein kinase C signaling
1.61	9.04 × 10^−6^	GO:0051023	Regulation of immunoglobulin secretion
1.61	9.04 × 10^−6^	GO:2000353	Positive regulation of endothelial cell apoptotic process
1.58	1.12 × 10^−5^	GO:0043089	Positive regulation of Cdc42 GTPase activity
1.58	1.12 × 10^−5^	GO:0045830	Positive regulation of isotype switching
1.55	3.49 × 10^−12^	GO:0021955	Central nervous system neuron axonogenesis
1.54	1.68 × 10^−5^	GO:0042510	Regulation of tyrosine phosphorylation of Stat1 protein
1.54	1.68 × 10^−5^	GO:0045911	Positive regulation of DNA recombination
1.37	8.84 × 10^−8^	GO:0048675	Axon extension
1.35	1.16 × 10^−7^	GO:0001890	Placenta development
1.33	1.70 × 10^−7^	GO:1990138	Neuron projection extension
1.32	1.52 × 10^−6^	GO:0000794	Condensed nuclear chromosome
1.32	1.52 × 10^−6^	GO:0071514	Genetic imprinting
1.22	7.83 × 10^−7^	GO:0048813	Dendrite morphogenesis
1.18	8.65 × 10^−6^	GO:0005720	Nuclear heterochromatin
1.13	2.89 × 10^−6^	GO:0048588	Developmental cell growth
1.11	1.94 × 10^−5^	GO:0000793	Condensed chromosome
1.08	2.35 × 10^−7^	GO:0060560	Developmental growth involved in morphogenesis
1.02	3.26 × 10^−6^	GO:0030900	Forebrain development

Logarithm of the odds ratio (LOD) larger than 1 were set as the cut-off criterion.

**Table A2:** Gene function annotation list of down-regulated gene sets in Colon-26MGS, but not in Colon-26HM4.

LOD	*p*-Value	GO ID	GO Name
2.97	2.62 × 10^−17^	GO:0003065	Positive regulation of heart rate by epinephrine
2.49	1.08 × 10^−5^	GO:0031630	Regulation of synaptic vesicle fusion to presynaptic membrane
2.49	1.08 × 10^−5^	GO:0046108	Uridine metabolic process
2.29	7.85 × 10^−10^	GO:0043402	Glucocorticoid mediated signaling pathway
2.28	1.66 × 10^−15^	GO:0003062	Regulation of heart rate by chemical signal
2.13	7.06 × 10^−15^	GO:0005862	Muscle thin filament tropomyosin
2.13	1.16 × 10^−6^	GO:0038062	Protein tyrosine kinase collagen receptor activity
2.13	1.16 × 10^−6^	GO:0038063	Collagen-activated tyrosine kinase receptor signaling pathway
2.13	1.16 × 10^−6^	GO:0038064	Collagen receptor activity
2.13	1.16 × 10^−6^	GO:0038065	Collagen-activated signaling pathway
2.13	1.16 × 10^−6^	GO:0061302	Smooth muscle cell-matrix adhesion
1.98	2.84 × 10^−10^	GO:0043423	3-phosphoinositide-dependent protein kinase binding
1.76	7.86 × 10^−6^	GO:0004886	9-*cis* retinoic acid receptor activity
1.72	4.80 × 10^−8^	GO:0090051	Negative regulation of cell migration involved in sprouting angiogenesis
1.65	9.42 × 10^−8^	GO:0010832	Negative regulation of myotube differentiation
1.59	1.72 × 10^−7^	GO:0006883	Cellular sodium ion homeostasis
1.52	3.88 × 10^−14^	GO:0031529	Ruffle organization
1.51	3.62 × 10^−6^	GO:0071498	Cellular response to fluid shear stress
1.50	4.02 × 10^−8^	GO:0010761	Fibroblast migration
1.48	6.35 × 10^−11^	GO:0030049	Muscle filament sliding
1.44	7.57 × 10^−7^	GO:0001553	Luteinization
1.44	7.57 × 10^−7^	GO:0007019	Microtubule depolymerization
1.38	3.59 × 10^−10^	GO:0033275	Actin-myosin filament sliding
1.37	1.69 × 10^−6^	GO:0048156	Tau protein binding
1.33	2.42 × 10^−6^	GO:0031115	Negative regulation of microtubule polymerization
1.33	2.42 × 10^−6^	GO:0061333	Renal tubule morphogenesis
1.33	2.42 × 10^−6^	GO:2000300	Regulation of synaptic vesicle exocytosis
1.31	4.51 × 10^−7^	GO:0034405	Response to fluid shear stress
1.30	1.53 × 10^−9^	GO:0010460	Positive regulation of heart rate
1.26	8.51 × 10^−7^	GO:0008585	Female gonad development
1.26	8.51 × 10^−7^	GO:0031109	Microtubule polymerization or depolymerization
1.24	6.30 × 10^−6^	GO:0014812	Muscle cell migration
1.24	6.30 × 10^−6^	GO:0051154	Negative regulation of striated muscle cell differentiation
1.24	6.30 × 10^−6^	GO:0055078	Sodium ion homeostasis
1.24	6.30 × 10^−6^	GO:1902803	Regulation of synaptic vesicle transport
1.21	1.51 × 10^−6^	GO:0043537	Negative regulation of blood vessel endothelial cell migration
1.21	1.51 × 10^−6^	GO:0071384	Cellular response to corticosteroid stimulus
1.19	1.22 × 10^−8^	GO:0045823	Positive regulation of heart contraction
1.19	2.26 × 10^−9^	GO:0070252	Actin-mediated cell contraction
1.15	2.60 × 10^−8^	GO:0061001	Regulation of dendritic spine morphogenesis
1.13	1.45 × 10^−9^	GO:0032781	Positive regulation of ATPase activity
1.11	2.29 × 10^−9^	GO:0051496	Positive regulation of stress fiber assembly
1.10	1.49 × 10^−6^	GO:0031113	Regulation of microtubule polymerization
1.06	6.60 × 10^−9^	GO:0032233	Positive regulation of actin filament bundle assembly
1.05	2.75 × 10^−6^	GO:0050775	Positive regulation of dendrite morphogenesis
1.04	2.78 × 10^−9^	GO:0004712	Protein serine/threonine/tyrosine kinase activity
1.03	1.43 × 10^−8^	GO:0030016	Myofibril
1.01	5.79 × 10^−9^	GO:0043462	Regulation of ATPase activity

Logarithm of the odds ratio (LOD) larger than 1 were set as the cut-off criterion.

**Table A3:** Gene expression of in vitro cultured cells by qRT-PCR.

Gene Name	ΔC_T_ of Dunn (Normalized by *ARP*)	ΔC_T_ of LM8 (Normalized by *ARP*)	Fold Change (LM8)	Significance
**Metastasis-related genes**				
*MMP2*	9.54 ± 0.01	6.99 ± 0.23	5.86	*
*MMP9*	15.57 ± 0.97	14.86 ± 1.31	1.64	NS
*VEGFA*	4.18 ± 0.04	4.70 ± 0.22	0.70	*
*PGF*	6.74 ± 0.06	5.75 ± 0.25	1.99	*
*FN1*	3.15 ± 0.03	6.51 ± 0.23	0.10	*
**Immune-related genes**				
*CD40*	9.25 ± 0.05	9.34 ± 0.34	0.94	NS
*CD11c*	ND	ND	ND	NS
*CD80*	16.36 ± 0.17	14.87 ± 0.06	2.82	*
*PDL1*	14.01 ± 0.65	14.01 ± 0.44	1.00	NS

Dunn or LM8 in vitro cultured cells were collected for qRT-PCR. NS: no significant; *: *p* < 0.05; ND: not detected.

**Table A4:** Gene expression of transplanted tumors by qRT-PCR.

Gene name	ΔC_T_ of Colon-26 (Normalized by *ARP*)	ΔC_T_ of Colon-26MGS (Normalized by *ARP*)	Fold change (Colon-26MGS)
**Metastasis-related genes**			
*MMP2*	9.99 ± 1.51	8.97 ± 1.14	2.03
*MMP9*	9.00 ± 1.01	7.19 ± 0.66	3.52
**Immune-related genes**			
*CD40*	10.23 ± 0.69	9.22 ± 0.31	2.02
*CD4*	15.05 ± 1.51	13.07 ± 0.42	3.94
*CD8a*	9.27 ± 0.40	8.01 ± 0.45	2.40

Colon-26 or Colon-26MGS cells were transplanted into the right hind leg, and two weeks later, the solid tumor sample was collected for qRT-PCR.

**Table A5:** PCR primers used in this study.

Gene Name	Product Size (bp)	Direction *	Sequence 5′–3′
*ARP*	193	F	TGCACTCTCGCTTTCTGGAGGGTGT
		R	AATGCAGATGGATCAGCCAGGAAGG
*GAPDH*	155	F	GGTGTGAACGGATTTGGCCGTATTG
		R	CCGTTGAATTTGCCGTGAGTGGAGT
*HPRT*	158	F	CAACGGGGGACATAAAAGTTATTGGTGGA
		R	TGCAACCTTAACCATTTTGGGGCTGT
*MMP2*	148	F	GTTGCCCCCTGATGTCCAGCAAGTA
		R	GGAGTCTGCGATGAGCTTAGGGAAACC
*MMP9*	147	F	CCAGAGCGTCATTCGCGTGGATAAG
		R	TGGTCCACCTTGTTCACCTCATTTTGG
*VEGFA*	93	F	GCACCCACGACAGAAGGAGAGCAGA
		R	CAGGGTCTCAATCGGACGGCAGTAG
*PGF*	113	F	GGAGACGACAAAGGCAGAAAGGAGGA
		R	AGTGGCTGGTTACCTCCGGGAAATG
*FN1*	105	F	CTGGAGGCAAACCCTGACACTGGAG
		R	CTGCCCGTTCGTGGGGGTAGTAGTT
*CD40*	124	F	ATCTCGCCCTGCGATGGTGTCTTT
		R	GCACTGGCCATCGTGGAGGTACTGT
*CD11c*	135	F	TCACAACCCCGTCCCTCTTATCGTG
		R	TGTCCATTTGCTTCCTCCAACATCTCC
*CD80*	145	F	CACTTGTGCTCTTTGGGGCAGGATT
		R	GGCCCGAAGGTAAGGCTGTTGTTTG
*PDL1*	125	F	TGCTAGATGTGGAGAAATGTGGCGTTG
		R	TTCACAGACCACAAGCTGCCAATCG
*S100a8*	149	F	CCATGCCCTCTACAAGAATGACTTCAAGAAA
		R	TCACCATCGCAAGGAACTCCTCGAA
*CD4*	145	F	ACCCCAGGTCTCGCTTCAGTTTGCT
		R	GGTAGGTCCCATCACCTCACAGGTCAA
*CD8a*	113	F	TTCAGTTCTGTCGTGCCAGTCCTTCA
		R	TCTGGTCTCTGGGGCTGAGATGTCC

* Direction of primer sequences: F = forward, R = reverse.
